# Development and analysis of a SmPC-based machine-readable dataset of contraindications for clinical decision support and real-world data analysis

**DOI:** 10.1007/s00228-026-04142-3

**Published:** 2026-07-16

**Authors:** Miriam Schechner, Wahram Andrikyan, Thomas Bauerdick, Theresa Terstegen, Louisa Redeker, Martin Federbusch, Katrin Farker, Alexander Strübing, Florian Schmidt, Petra Thürmann, Sven Schmiedl, Hanna M. Seidling, Tobias Dreischulte, Daniel Neumann, Markus Loeffler, Renke Maas, Ulrich Jaehde, Martin F. Fromm, Michael I. Sponfeldner

**Affiliations:** 1 Institute of General Practice and Family Medicine, LMU University Hospital, LMU Munich, Munich, Germany; 2 Institute of Experimental and Clinical Pharmacology and Toxicology, Friedrich-Alexander-Universität Erlangen-Nürnberg, Erlangen, Germany; 3 Universitätsklinikum Erlangen, Erlangen, Germany; 4 Department of Clinical Pharmacy, Institute of Pharmacy, University of Bonn, Bonn, Germany; 5 Medical Faculty Heidelberg, Internal Medicine IX, Clinical Pharmacology and Pharmacoepidemiology, Cooperation Unit Clinical Pharmacy, Heidelberg University, Heidelberg University Hospital, Heidelberg, Germany; 6 Department of Clinical Pharmacology, School of Medicine, Faculty of Health, Witten / Herdecke University, Wuppertal, Germany; 7 Department for Clinical AI and Translational Medicine, University of Leipzig Medical Center, Leipzig, Germany; 8 Institute of Laboratory Medicine, Clinical Chemistry and Molecular Diagnostics, University of Leipzig Medical Center, Leipzig, Germany; 9 Institute for Hospital Pharmacy, University Center for Pharmacotherapy and Pharmacoeconomics, Jena University Hospital, Jena, Germany; 10 Institute for Medical Informatics, Statistics and Epidemiology (IMISE), Leipzig University, Leipzig, Germany; 11 FAU NeW – Research Center New Bioactive Compounds, Friedrich- Alexander-Universität Erlangen-Nürnberg, Erlangen, Germany

**Keywords:** Contraindication, CDSS, Medication safety, Routine data

## Abstract

**Purpose:**

Unrecognized contraindications pose risks for adverse drug reactions, hospitalizations or death. Clinical decision support systems (CDSS) aim to mitigate medication-related harm, particularly originating from contraindications. However, many CDSS provide limited benefit, as they focus largely on singular risk situations such as drug-drug interactions and often generate alerts of limited clinical relevance. Comprehensive integration of contraindications into CDSS may support more clinically meaningful alerts. The aim of this work was the development and analysis of machine-readable contraindication lists, including drug-clinical condition, drug-kidney function and drug-drug (group) contraindications, for integration into CDSS and real-world data analysis.

**Methods:**

We extracted and operationalized contraindications, based on Summaries of Product Characteristics (SmPCs), of the 688 most prescribed drugs in Germany, leveraging common medical coding systems. Moreover, we analyzed extracted contraindications based on operationalizability, overall frequency and frequencies within different contraindication categories.

**Results:**

In total, we extracted 4676 contraindications, of which 2129 (45.5%) were deemed operationalizable. Of these 2129 contraindications, 1652 (77.6%) were attributed to drug-clinical condition, 83 (3.9%) to drug-kidney, 140 (6.6%) to drug-drug group and 254 (11.9%) to drug-drug. The most frequently mentioned contraindicated risk situations were ‘severe liver insufficiency’ (*n* = 74, 3.5%), ‘pregnancy’ (*n* = 66, 3.1%), ‘MAO-inhibitors’ (*n* = 44, 2.1%), and ‘shock’ (*n* = 44, 2.1%).

**Conclusion:**

Our results show, that drug-clinical condition contraindications are listed far more frequently in SmPCs than other contraindication categories. Focusing on clinical condition-related contraindications within CDSS could improve the detection of clinically relevant contraindications in routine data and enhance medication safety. The clinical applicability is currently being evaluated in the INTERPOLAR study.

**Supplementary Information:**

The online version contains supplementary material available at 10.1007/s00228-026-04142-3.

## Introduction

The European Medicines Agency (EMA) defines a contraindication as a situation in which a drug must not be given for safety reasons [1]. Such situations may include, for instance, clinical diagnoses, concomitantly administered drugs, age, sex or other patient-specific predispositions (e.g., genetic factors), where the risks of administering a drug outweigh its potential benefits.

Despite the fact that contraindications are among the most safety-critical medication risks and may result in adverse drug reactions, hospitalizations or even death, their reliable detection in routine clinical care remains limited [2–4]. Various strategies, such as clinical decision support systems (CDSS) embedded within the prescribing process, have been previously proposed and implemented in order to detect contraindications early and improve medication safety [5, 6]. In practice, however, many CDSS predominately focus on drug-drug interaction alerts or dosing recommendations in renal impairment while insufficiently addressing contraindicated combinations of drugs and clinical conditions (e.g., beta-blockers in patients with severe asthma) or drug administration despite severe laboratory abnormalities (e.g., furosemide in the presence of severe hypokalaemia) [5, 7, 8]. 

Moreover, a substantial proportion of CDSS-alerts is considered of questionable clinical relevance, which reduces their perceived usefulness, prompting clinicians to override alerts more frequently due to alert fatigue and gradually eroding trust in these systems [9, 10]. This leads to limited clinical usefulness of CDSS in terms of mitigating medication-related harm, despite their now widespread adoption. A meta-analysis of more than 1.2 million patients reported that use of CDSS increased the proportion of patients receiving recommended care by only 5.8% on average [11]. 

Focusing on contraindications may help increase the clinical relevance of CDSS-alerts, although this potential benefit remains uncertain due to the lack of evidence on their population-level prevalence, both in the in- and outpatient setting, as well as their impact on clinical outcomes. Generating such evidence warrants publicly available, machine-readable contraindication lists, which are currently lacking in literature. Achieving this, in turn, requires the systematic operationalization of contraindications, i.e., the translation of a clinical risk situation into a standardized, measurable data item that can be mapped to structured data within electronic health records.

Therefore, the INTERPOLAR study (INTERventional POLypharmacy-drug interActions-Risks, German Clinical Trials Register ID: DRKS00034730) aims to develop and test IT-based algorithms capable of identifying contraindications in clinical real-world data within the German hospital setting [12, 13]. Consequently, the present study aimed to (1) identify and operationalize contraindications of the most prescribed drugs in Germany and (2) analyze and categorize these contraindications according to their frequency of occurrence within the included Summaries of Product Characteristics (SmPCs). Thereby, we provide a structured, machine-readable dataset suitable for integration into CDSS and real-world data analysis of contraindication-related risks.

## Methods

The workflow for the development and characterization of a structured, machine-readable dataset of contraindications is described in the following. First, contraindications had to be defined, selected and extracted from German SmPCs as well as operationalized. The resulting contraindications were then characterized and analyzed.

### Definition of ‘contraindications’ in SmPCs

In this study, a contraindication was defined in accordance with the guidance for SmPCs provided by the EMA as situations in which a drug must not be given for safety reasons [1]. Accordingly, situations, in which a treatment or procedure should be used with caution but can be considered in cases of the anticipated benefit outweighing potential risks, were not considered contraindications.

For operationalization, a contraindication was then defined as a combination of drug and corresponding risk situation, that was classified into one of four different categories:

1. Drug-clinical condition, i.e., a combination of a drug and a clinical condition or diagnosis (e.g., ibuprofen and active bleeding),
2. Drug-kidney function, as a separated sub-category of drug-clinical condition, i.e., a combination of a drug and a certain degree of renal impairment (e.g., metformin and estimated glomerular filtration rate (eGFR) < 30 mL/min). Drug-kidney contraindications were separated from the general drug-clinical condition category, since renal impairment is frequently addressed independently in SmPCs and requires specific operationalization based on pre-defined kidney function thresholds.
3. Drug-drug group, i.e., a combination of a drug and a pharmacological class or group of drugs (e.g., simvastatin and cytochrome P450 (CYP) 3A4 inhibitors), and
4. Drug-drug, i.e., a combination of a drug and another individual drug (e.g., ciclosporin and dabigatran etexilate) as a separate category from drug-drug group, for contraindications regarding individual, additional drugs that were not attributed to any drug group within the SmPCs.

### Selection of SmPCs

**Fig. 1:**
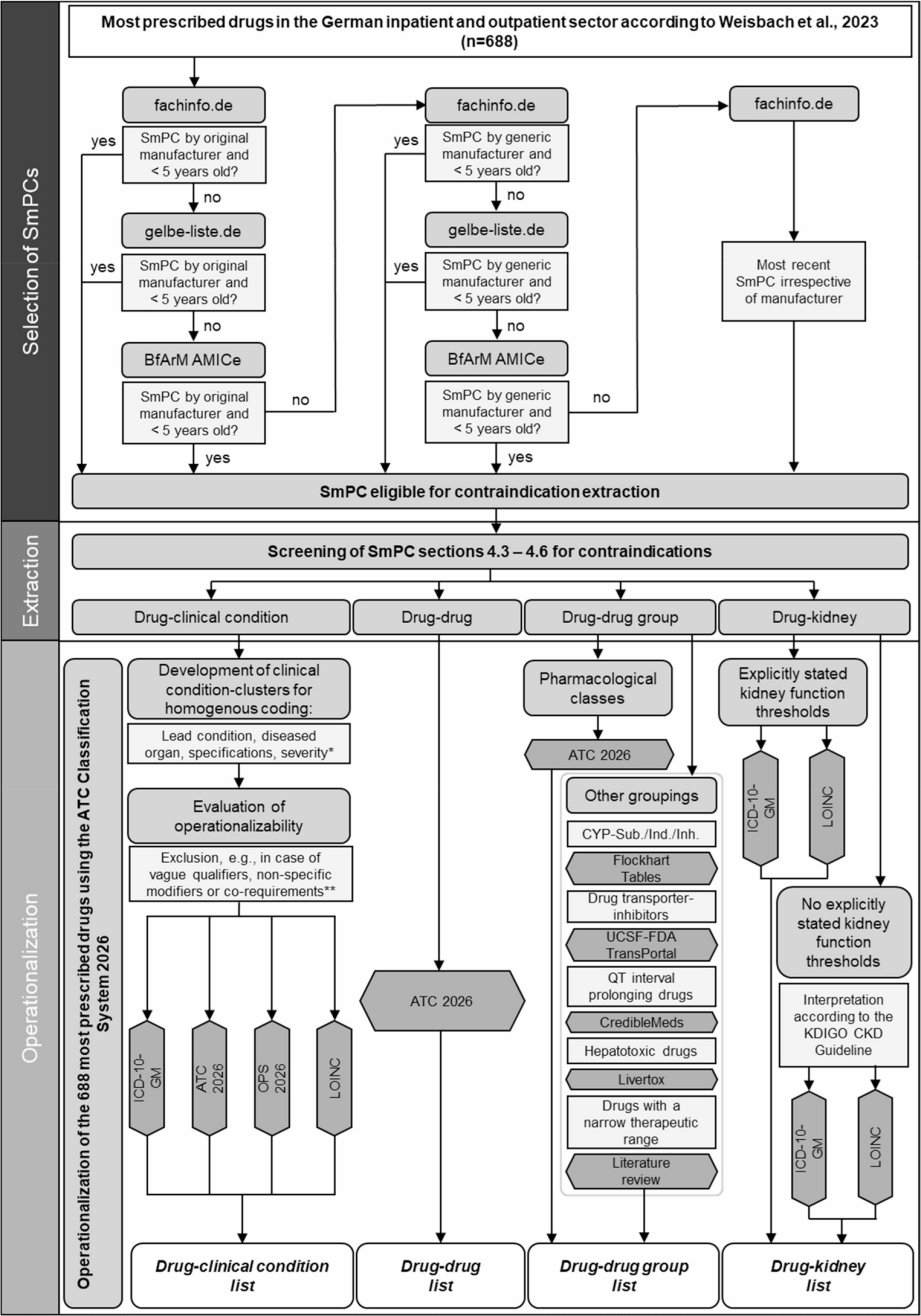
Workflow for the extraction and operationalization of contraindications based on Summaries of Product Characteristics. Legend: The list of the most prescribed drugs is based on Weisbach et al. [14] * = contraindications were standardized through a hierarchical clustering approach, starting with a ‘lead-condition’ (e.g., tumor), optionally followed by ‘diseased organ’ (e.g., tumor, breast), ‘specifications’ (e.g., tumor, breast, HER2 positive), and ‘severity’ (e.g., hyperkalaemia, severe) as far as applicable. ** = clinical conditions were deemed non-operationalizable if they were accompanied by vague qualifiers such as staging (e.g., ‘severe respiratory depression’) or non-specific modifiers (e.g., ‘clinically relevant’). Abbreviations: ATC = Anatomical Therapeutic Chemical; SmPC = Summary of Product Characteristics; ICD-10-GM = International Classification of Diseases, Version 10, German Modification; OPS = Operation and Procedure Codes; LOINC = Logical Observation Identifiers Names and Codes; CYP = Cytochrome P450 enzyme; Sub. = Substrate; Ind. = Inducer; Inh. = Inhibitor; KDIGO-CKD Guideline = Kidney Disease: Improving Global Outcomes – Chronic Kidney Disease Guideline.

The selection of drugs used for the following analyses is based on a prior study by Weisbach et al. [14], which identified the most prescribed drugs in the German outpatient and inpatient sector (*n* = 688). The detailed selection process of SmPCs is shown in Fig. 1 as well as provided in the supplementary methods.

### Extraction of contraindications from SmPCs

For extraction of contraindications, the SmPC Sect.  4.3 (‘contraindications’) was manually screened. To improve sensitivity, the Sect.  4.4. (‘special warnings and precautions for use’), 4.5. (‘interactions with other drugs and other interactions’), and 4.6. (‘fertility, pregnancy and breastfeeding’) were also screened, to identify contraindications that were placed in other SmPC sections. Subsequently to initial extraction by one researcher, the contraindications were validated by independent extraction of the SmPCs and verification by another researcher. Following extraction, the identified contraindications were categorized into the previously described classification system: (1) Drug-clinical condition, (2) Drug-kidney, (3) Drug-drug group, and (4) Drug-drug (Fig. 1).

### Operationalization of contraindications within different contraindication categories

The selection of medical coding systems within the contraindication categories were based on the Core Data Set of the German Medical Informatics Initiative and its interoperability specifications within which the INTERPOLAR study and this work were conducted [12, 13]. In addition, operationalizability was assessed within the context of the INTERPOLAR study, according to the categories detailed below. Highly complex contraindications, such as those whose operationalization would have required additional expert consensus, e.g., ‘clinically relevant hypertension’, were beyond the scope of this work. The process of operationalization of the Anatomical Therapeutic Chemical (ATC) codes of the 688 most prescribed drugs in Germany is described in the supplementary methods. Technical specifications regarding machine readability and validation are also provided in the supplementary methods.

#### Drug-clinical condition contraindications

##### Modification and editing of the drug-clinical condition contraindication list

Prior to the operationalization of contraindications within the drug-clinical condition contraindication list, the extracted list was systematically refined to ensure comprehensive coverage and enable consistency: First, clinical conditions that were mentioned as groups in SmPCs were disaggregated into individual contraindications, e.g., ‘arrhythmias such as Torsades de Pointes (TdP) or atrioventricular (AV)-block’ to ‘TdP’ and ‘AV-block’. Second, to address the variability in phrasing of contraindications that occurred in multiple SmPCs, the contraindications were harmonized through a hierarchical clustering approach, starting with a ‘lead-condition’ (e.g., tumor), optionally followed by ‘diseased organ’ (e.g., tumor, breast), ‘specifications’ (e.g., tumor, breast, HER2 positive), and ‘severity’ (e.g., hyperkalaemia, severe) as far as applicable. This approach ensured that all relevant attributes of the clinical condition were retained. Each individual step of the entire editing and standardization process of the drug-clinical condition contraindication list was independently reviewed and validated by at least two of five researchers.

##### Operationalizability of drug-clinical condition contraindications

As a general rule for operationalization of drug-clinical condition contraindications, clinical conditions were only considered operationalizable, if they could be unequivocally mapped to the coding systems selected for operationalization. For instance, if a SmPC listed a clinical condition with examples such as ‘arrhythmias including TdP or AV-block’, only the explicitly named examples were operationalized. Furthermore, clinical conditions were deemed non-operationalizable if they were accompanied by non-specific modifiers such as ‘clinically relevant’. In addition, contraindications that required contextual qualifiers such as specific routes of administration (e.g., intramuscular) or had time constraints were also considered non-operationalizable. The rationale behind this restrictive approach was to ensure very high specificity and thus a low rate of false positive signals when applying the list in CDSS or for analysis of real-world data. Dose-dependent contraindications were excluded from this analysis. In cases where a mild or moderate severity stage of a condition was specified in the respective SmPC, e.g., ‘moderate renal insufficiency’, more severe stages were also included in the operationalization.

##### Operationalization of drug-clinical condition contraindications

For operationalization of drug-clinical condition contraindications, the following coding systems and methodologies were used:

1. International Classification of Diseases, Version 10, German Modification (ICD-10-GM) for coding of clinical conditions. ICD-10 codes were considered appropriate, if they were specific, i.e., only included the clinical condition in question. ICD-10 codes were retrieved from kbv.de [15].
2. ATC, Version 2026 for coding of clinical conditions through drugs that are unequivocally and solely used for treatment of the clinical condition in question, e.g., ‘roflumilast’ for ‘chronic obstructive pulmonary disease’. ATC Codes were retrieved from BfArM.de [16].
3. Operation and Procedure (OPS) Codes, Version 2026 for coding of clinical conditions through operations and procedures, e.g., ‘surgical interventions on the central nervous system’. OPS codes were retrieved from BfArM.de [17].
4. Logical Observation Identifiers Names and Codes (LOINC) for coding of clinical conditions through specific laboratory parameters, e.g., for the clinical condition ‘hyperkalaemia’. For the detailed methodology of LOINC coding see supplementary methods.

All steps of operationalization were conducted independently by two researchers and compared. In cases of disagreement, a third researcher was consulted to decide upon operationalizability and appropriate coding.

#### Drug-kidney contraindications

##### Interpretation of kidney function thresholds

For the operationalization of the drug-kidney contraindication list as a subgroup of the general drug-clinical condition category, thresholds for kidney functions that constituted a contraindication were extracted from the SmPCs. If explicit numerical thresholds (e.g., eGFR < 30 mL/min) were stated in the respective SmPC, they were used for operationalization. In cases of terms such as of ‘mild kidney insufficiency’, ‘moderate kidney insufficiency’, ’severe kidney insufficiency’ or ‘terminal kidney insufficiency’ stated in the SmPC, we referred to the eGFR thresholds of < 90 mL/min, < 60 mL/min, < 30 mL/min, and < 15 mL/min, respectively, established by the KDIGO Chronic Kidney Disease (CKD) Guideline [18]. If a SmPC used the term ‘kidney failure’ the eGFR threshold of < 15 mL/min was used. ‘Acute kidney injury (AKI)’ stated in the SmPC, was not operationalized by means of an explicit eGFR threshold, as this clinical condition cannot be defined by a single eGFR value; instead, the corresponding ICD-10-GM code was used.

##### Operationalization of the drug-kidney contraindication list

In general, the operationalization was performed based on both, ICD-10-GM codes representing AKI or CKD stages as well as LOINC codes corresponding to eGFR or creatinine clearance (CrCl), depending on the method mentioned in the SmPC. Relevant cut-off values were applied to laboratory parameters to estimate kidney function and map it to the defined thresholds. LOINC codes were operationalized as described in the supplementary methods.

#### Drug-drug group contraindications

For operationalization of the drug-drug group contraindication list, the ATC Version 2026 (BfArM.de [16]) was used to define and code pharmacological drug classes such as ‘macrolide antibiotics’. Next to pharmacological drug classes, different other drug groups were identified within the SmPCs: (1) Substrates / Inducers / Inhibitors of CYP enzymes which were defined based on the classification of the Flockhart Table [19], (2) QT interval prolonging drugs, defined based on the classification in CredibleMeds [20], (3) inhibitors of transport proteins, defined based on the UCSF-FDA TransPortal database [21], (4) drugs with a narrow therapeutic range were identified based on a literature review [22], (5) hepatotoxic drugs were operationalized using LiverTox [23], and (6) drugs known to cause agranulocytosis were operationalized as in a study by Wermund et al. [24]. The operationalization of drug-drug group contraindications is described in the supplementary methods.

#### Drug-drug contraindications

Similarly, for operationalization of the drug-drug contraindication list, the corresponding individual drugs listed as contraindications in the SmPCs were coded using ATC Version 2026 (BfArM.de [16]), as described in the supplementary methods.

### Analysis of non-operationalizable contraindicated risk situations

The contraindicated risk situations considered non-operationalizable were analyzed and categorized according to categories such as ‘hypersensitivity to the respective drug’, ‘non-operationalizable due to additional conditions’, or ‘no suitable coding options available’. This step was performed by two researchers individually and then compared. Cases of disagreement were discussed and resolved.

### Analysis of extracted and operationalized risk situations

Extracted and operationalized risk situations were analyzed using descriptive statistics, i.e., categorical summarization as well as absolute and relative frequency counts. We first determined the proportion of contraindications that could be operationalized according to the criteria outlined above. The most frequently listed risk situations in SmPCs within each category were then identified based on the used coding systems – ICD-10-GM for drug-clinical condition and ATC for drug-drug group and drug-drug. For drug-kidney we identified the groups of drugs (ATC level 2) that were most commonly contraindicated in patients with renal insufficiency. Furthermore, we categorized the most frequently listed kidney function thresholds.

## Results

### Characterization of the most prescribed drugs in Germany

The characterization of the 688 most prescribed drugs in the German inpatient and outpatient sector, as identified by Weisbach et al. [14], according to the ATC Classification 2026 is provided in the Supplementary Material (supplementary methods and Figure S1).

### Selection of SmPCs and extraction of contraindications

**Fig. 2:**
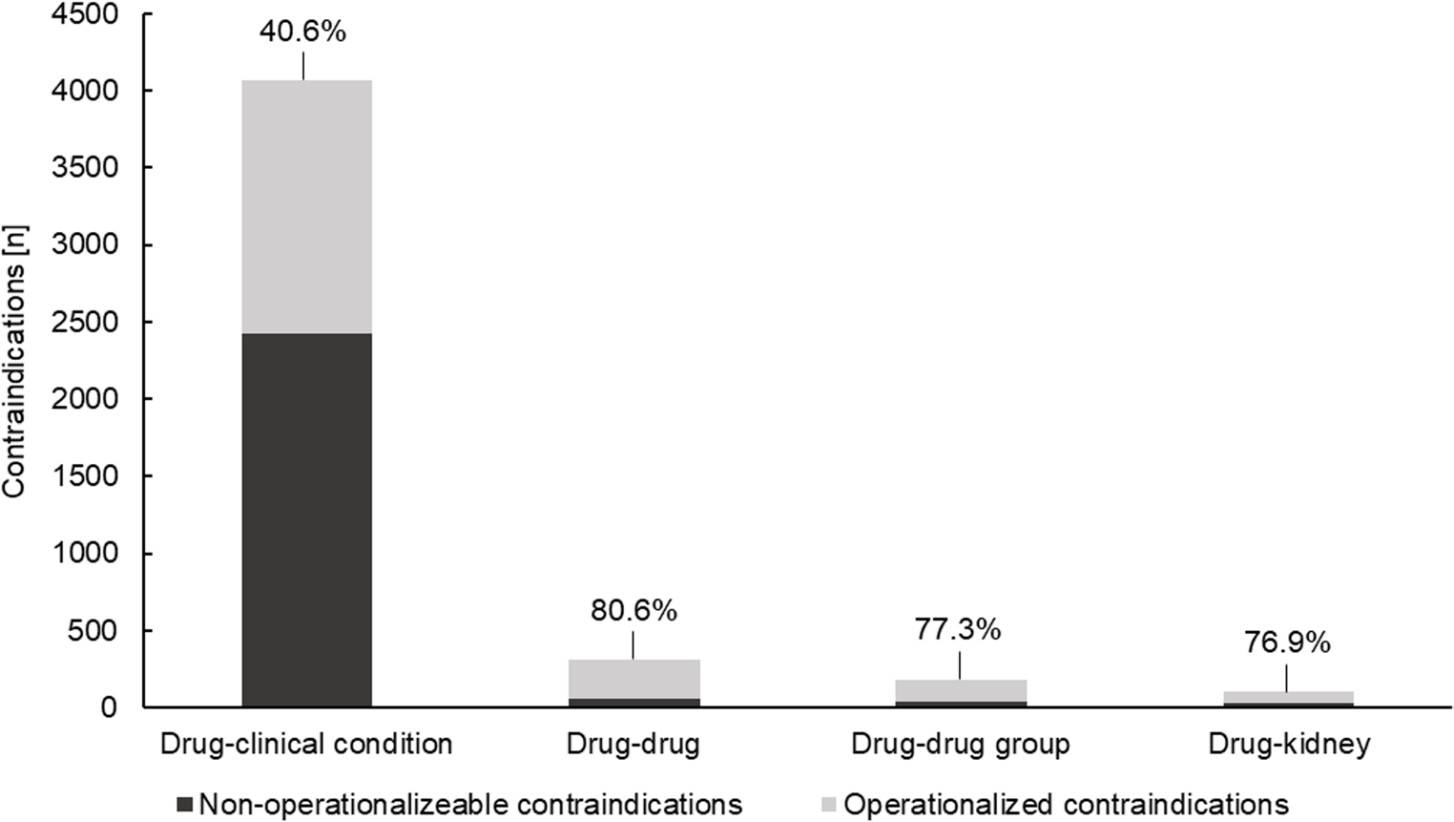
Composition of extracted and operationalized contraindications. Legend: Numbers of contraindications extracted from the SmPCs of the 688 most prescribed drugs in Germany and the final, operationalized lists of contraindications, categorized by drug-clinical condition, drug-kidney, drug-drug group and drug-drug. The percentage accounts for the proportion of operationalized contraindications within each category

Screening yielded a total of 4676 individual contraindications, i.e., combinations of drugs and corresponding risk situations (e.g., apixaban and gastrointestinal bleeding) with a median of 5 contraindicated risk situations per SmPC (IQR 2 to 10). Of these, 4072 (87.1%) contraindications were classified as drug-clinical condition with 108 additional (2.3%) contraindications classified as drug-kidney, 181 (3.9%) contraindications were classified as drug-drug group and 315 (6.7%) contraindications were classified as drug-drug (Fig. 2). Following extraction and classification, 2547 (54.5%) contraindicated risk situations were considered non-operationalizable and were therefore subsequently excluded to ensure high specificity.

### Operationalization of contraindications

**Fig. 3:**
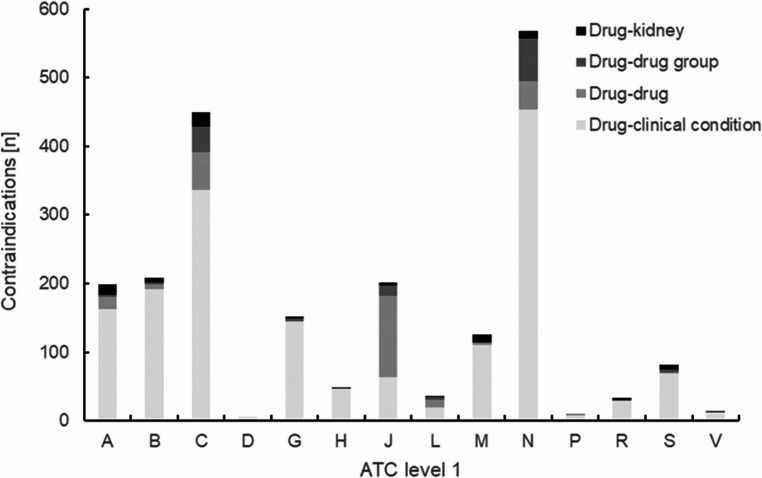
Characterization of contraindications of the 688 most prescribed drugs in Germany by Anatomical Therapeutic Chemical (ATC) classification system. Legend: Characterization of the frequencies of contraindications based on ATC level 1. The list of the most prescribed drugs is based on Weisbach et al. [14]. Abbreviations: ATC = Anatomical Therapeutic Chemical; A = Alimentary tract and metabolism; B = Blood and blood forming organs; C = Cardiovascular system; D = Dermatologicals; G = Genito urinary system and sex hormones; H = Systemic hormonal preparations, excl. sex hormones and insulins; J = Antiinfective for systemic use; L = Antineoplastic and immunomodulating agents; M = Musculo-skeletal system; N = Nervous system; P = Antiparasitic products, insecticides and repellents; R = Respiratory system; S = Sensory organs; V = Various

A total of 2129 (45.5%) contraindications were operationalized utilizing commonly used coding systems in Germany (ATC, ICD-10, LOINC, OPS), of which 1652 (77.6%) contraindications were classified as drug-clinical condition, 83 (3.9%) contraindications as drug-kidney, 140 (6.6%) contraindications as drug-drug group and 254 (11.9%) additional contraindications as drug-drug, since they were not mentioned with respect to a drug group within the SmPCs (Fig. 2). According to the ATC classification of the 688 most prescribed drugs, drugs attributable to the ATC level 1 categories ‘nervous system’, ‘cardiovascular system’, and ‘blood and blood forming organs’ had the highest number of contraindicated risk situations, i.e., 568 (26.7%), 449 (21.1%) and 208 (9.8%), listed in their SmPCs, respectively (Fig. 3). Within these ATC level 1 categories, the most frequently listed contraindications were ‘alcohol intoxication’, ‘severe liver insufficiency’, and ‘coma’ for drugs in the category ‘nervous system’; ‘shock’, ‘pregnancy, general’, and ‘pregnancy, 2^nd^ and 3^rd^ trimester’ for drugs in the category ‘cardiovascular system’; and ‘gastrointestinal ulcers’, ‘intracranial bleeding’, and ‘aneurysm’ for drugs in the category ‘blood and blood forming organs’.

Overall, the most frequently listed contraindicated risk situations were ‘severe liver insufficiency’ (*n* = 74, 3.5%), followed by ‘pregnancy’ (*n* = 66, 3.1%), ‘monoamine oxidase (MAO)-inhibitors’ (*n *= 44, 2.1%), and ‘shock’ (*n* = 44, 2.1%). The ten most frequently listed and operationalized contraindicated risk situations across all four categories are listed in Table 1.

**Table 1:** Top 10 most frequently listed contraindicated risk situations in SmPCs of the 688 most prescribed drugs in Germany

Contraindicated risk situation	*n* (% of all operationalized contraindications)
Severe liver insufficiency	74 (3.5)
Pregnancy	66 (3.1)
Shock	44 (2.1)
Monoamine oxidase (MAO)-inhibitors	44 (2.1)
Acute myocardial infarction	38 (1.8)
Alcohol intoxication	34 (1.6)
Ileus	31 (1.5)
Gastrointestinal ulcers	27 (1.3)
Myasthenia gravis	27 (1.3)
Acute heart failure	22 (1.0)

#### Drug-clinical condition

Of the 1652 drug-clinical condition contraindications operationalized, the most frequently listed contraindicated clinical conditions were attributable to the ICD-10-GM categories ‘diseases of the circulatory system’ (*n* = 373, 22.6%), followed by ‘diseases of the digestive system’ (*n* = 273, 16.5%) and ‘endocrine, nutritional and metabolic diseases’ (*n* = 185, 11.2%) (Table 2). Overall, the most frequently listed contraindicated clinical conditions were ‘severe liver insufficiency’ (*n* = 74, 4.5%), ‘pregnancy’ (*n *= 66, 4.0%), and ‘shock’ (*n *= 44, 2.7%). Concerning the coding systems used for operationalization of clinical conditions, ICD-10-GM codes were assigned to 1544 (93.5%) conditions, ATC codes were assigned to 147 (8.9%), OPS codes were assigned to 24 (1.5%), and LOINC codes were assigned to 497 (30.1%) clinical conditions, with the possibility of clinical conditions getting assigned codes from multiple coding systems. In total, the ten most frequently listed contraindicated clinical conditions accounted for 23.2% of all operationalized drug-clinical condition contraindications and 9.4% of all extracted drug-clinical condition contraindications.

**Table 2:** Most frequent contraindicated clinical conditions listed in SmPCs of the 688 most prescribed drugs in Germany classified based on the International Classification of Diseases, Version 10, German Modification (ICD-10-GM)

ICD-10-GM chapter	n*	Most frequent contraindicated clinical conditions (*n* [% of all contraindicated clinical conditions attributed to the respective ICD-10-GM chapter])
(I) Certain infections and parasitic diseases	25	Ophthalmic tuberculosis (5 [20.0]) Herpes simplex keratitis (5 [20.0]) Acute hepatitis (4 [16.0])
(II) Neoplasms	50	Phaeochromocytoma (21 [42.0]) Malignant breast tumor (8 [16.0]) Malignant endometrial tumor (5 [10.0])
(III) Diseases of the blood and blood-forming organs and certain disorders involving the immune mechanism	99	Anaemia (19 [19.2]) Active bleeding (9 [9.1]) Haemorrhagic diathesis (9 [9.1])
(IV) Endocrine, nutritional and metabolic diseases	185	Hypokalaemia (20 [10.8]) Hypovolaemia (16 [8.6]) Hypercalcaemia (15 [8.1])
(V) Mental and behavioural disorders	53	Alcohol dependence (10 [18.9]) Substance dependence (8 [15.1]) Drug dependence (8 [15.1])
(VI) Diseases of the nervous system	101	Myasthenia gravis (27 [26.7]) Sleep apnoea syndrome (11 [10.9]) Epilepsy (7 [6.9])
(VII) Diseases of the eye and adnexa	56	Angle-closure glaucoma (17 [30.4]) Glaucoma (8 [14.3]) Eye surgery (8 [14.3])
(VIII) Diseases of the ear and mastoid process	2	Tinnitus (1 [50.0]) Ear surgery (1 [50.0])
(IX) Diseases of the circulatory system	373	Acute myocardial infarction (38 [10.2]) Acute heart failure (22 [5.9]) Sick sinus syndrome (17 [4.6])
(X) Diseases of the respiratory system	72	Bronchial asthma (16 [22.2]) Respiratory insufficiency (12 [16.4]) Severe chronic obstructive pulmonary disease (10 [13.7])
(XI) Diseases of the digestive system	273	Severe liver insufficiency (74 [27.1]) Ileus (31 [11.4]) Gastrointestinal ulcers (27 [9.9])
(XII) Diseases of the skin and subcutaneous tissue	10	Erythema exsudativum multiforme (3 [30.0]) Toxic epidermal necrolysis (2 [20.0]) Rosacea (2 [20.0])
(XIII) Diseases of the musculoskeletal system and connective tissue	9	Gout (5 [55.6]) Systemic lupus erythematosus (1 [11.1]) Osteomyelitis (1 [11.1])
(XIV) Diseases of the genitourinary system	22	Nephrolithiasis (4 [18.2]) Glomerulonephritis (4 [18.2]) Urinary tract stenosis (3 [13.6])
(XV) Pregnancy, childbirth and the puerperium	141	Pregnancy (66 [46.8]) Pregnancy, 2^nd^ and 3^rd^ trimester (18 [12.8]) Pregnancy, 3^rd^ trimester (13 [9.2])
(XVI) Congenital malformations, deformations and chromosomal abnormalities	6	Arteriovenous malformation (5 [83.3]) Aortic isthmus stenosis (1 [16.7])
(XVII) Symptoms, signs and abnormal clinical and laboratory findings, not elsewhere classified	111	Shock (44 [39.6]) Coma (18 [16.2]) Bradycardia (10 [9.0])
(XVIII) Injury, poisoning and certain other consequences of external causes	59	Alcohol intoxication (34 [57.6]) Angiooedema (11 [18.6]) Traumatic brain injury (4 [6.8])
(XIX) Factors influencing health status and contact with health services	5	Drug substitution (2 [40.0]) Organ transplantation (1 [20.0]) Radiotherapy (1 [20.0])
Total	1652	

*absolute number of contraindicated clinical conditions of the respective ICD-10-GM Chapter

#### Drug-kidney

Of the 83 drug-kidney contraindications operationalized, the drug groups most frequently contraindicated based on kidney function were ‘diuretics’ (*n* = 9, 10.8%), ‘mineral supplements’ (*n* = 7, 8.4%), and ‘anti-inflammatory and anti-rheumatic products’ (*n* = 7, 8.4%) (Table 3). In 44 (53.0%) cases, contraindications were based on eGFR thresholds and in 35 (42.2%) cases contraindications were based on CrCl thresholds. ‘eGFR < 30 mL/min’ was the most frequently listed eGFR threshold (*n* = 27, 32.5%) and ‘CrCl < 30 mL/min’ was the most frequently listed CrCl threshold (*n* = 27, 32.5%).

**Table 3:** Drug groups of the 688 most prescribed drugs in Germany with contraindications regarding renal insufficiency listed in the SmPCs, classified based on Anatomical Therapeutic Chemical (ATC) classification system level 2

ATC chapter (Level 2)	*n* (% of all drugs with listed drug-kidney contraindications)
C03 - Diuretics	9 (10.8)
A12 - Mineral supplements	7 (8.4)
M01 - Anti-inflammatory and anti-rheumatic products	7 (8.4)
S01 - Ophthalmologicals	6 (7.2)
A10 - Drugs used in diabetes	4 (4.8)
J01 - Antibacterials for systemic use	4 (4.8)
N02 - Analgesics	4 (4.8)
C10 - Lipid modifying agents	3 (3.6)
B01 - Antithrombotic agents	3 (3.6)
B05 - Blood substitutes and perfusion solutions	3 (3.6)
C01 - Cardiac therapy	3 (3.6)
C09 - Agents acting on the renin-angiotensin systems	3 (3.6)
A07 - Antidiarrheals, intestinal anti-inflammatory / antiinfective agents	2 (2.4)
G04 - Urologicals	2 (2.4)
M03 - Muscle relaxants	2 (2.4)
N05 - Psycholeptics	2 (2.4)
N06 - Psychoanaleptics	2 (2.4)
Other	17 (20.5)

#### Drug-drug group

Of the 140 drug-drug group contraindications operationalized, the most frequently contraindicated drug groups were attributable to the ATC chapters ‘nervous system’ (*n* = 64, 45.7%) followed by ‘cardiovascular system’ (*n* = 14, 10.0%). Additionally, the drug groups ‘CYP Substrates / Inducers / Inhibitors’, ‘QT interval prolonging drugs’, and ‘drugs with a narrow therapeutic range’ accounted for 24.3% (*n* = 34) of the drug-drug group contraindications (Table 4). In total, the ten most frequently listed contraindicated drug groups accounted for 75.0% of all operationalized drug-drug group contraindications and 58.0% of all extracted drug-drug group contraindications.

**Table 4:** Most frequently listed contraindicated drug groups in SmPCs of the 688 most prescribed drugs in Germany classified based on Anatomical Therapeutic Chemical (ATC) classification system

ATC chapter	*n*	Most frequent contraindicated drug groups (*n* [% of all contraindicated drug groups attributed to the respective ATC chapter])
A – Alimentary tract and metabolism	4	Antacids (1 [25.0]) H_2_ Antihistamines (1 [25.0]) Proton pump inhibitors (1 [25.0])
B – Blood and blood forming organs	1	Thrombolytics (1 [100.0])
C – Cardiovascular system	14	Antiarrhythmics (4 [28.6]) Guanylate cyclase activators (3 [21.4]) ACE inhibitors (2 [14.3])
D – Dermatologicals	2	Psoralens (1 [50.0]) Retinoids (1 [50.0])
G – Genito-urinary system and sex hormones	7	Phosphodiesterase-5 inhibitors (6 [85.7]) Alpha-1 blockers (1 [14.3])
J – Antiinfectives for systemic use	6	Aminoglycosides (1 [16.7]) Cephalosporins (1 [16.7]) Tetracyclines (1 [16.7])
L – Antineoplastic and immunomodulating agents	2	5-Fluorouracil prodrugs (1 [50.0]) Immunosuppressants (1 [50.0])
M – Musculo-skeletal systems	1	Depolarizing muscle relaxants (1 [100.0])
N – Nervous systems	64	MAO inhibitors (44 [68.8]) Ergotamin derivatives (8 [12.5]) Triptans (4 [6.3])
R – Respiratory system	1	Indirect acting sympathomimetics (1 [100.0])
V – Various	1	Iron chelators (1 [100.0])
Miscellaneous	37	CYP Substrates / Inducers / Inhibitors (17 [45.9]) QT interval prolonging drugs (15 [40.5]) Drugs with a narrow therapeutic range (2 [5.4])
Total	140	

#### Drug-drug

Of the 254 drug-drug contraindications operationalized, the most frequently contraindicated drugs were attributable to the ATC chapters ‘antiinfectives for systemic use’ (*n* = 72, 28.3%), followed by ‘cardiovascular system’ (*n* = 61, 24.0%), and ‘nervous system’ (*n* = 47, 18.5%). Overall, the most frequently listed contraindicated drugs were ‘pimozide’ (*n* = 8, 3.1%), ‘ciclosporin’ (*n* = 8, 3.1%) and ‘valsartan with sacubitril’ (*n* = 7, 2.8%) (Table 5). In total, the ten most frequently listed contraindicated drugs accounted for 24.0% of all operationalized drug-drug contraindications and 19.4% of all extracted drug-drug contraindications.

**Table 5:** Most frequently listed contraindicated drugs in SmPCs of the 688 most prescribed drugs in Germany classified based on Anatomical Therapeutic Chemical (ATC) classification system

ATC chapter	*n*	Most frequent contraindicated drugs (*n* [% of all contraindicated drugs attributed to the respective ATC chapter])
A – Alimentary tract and metabolism	15	Cisapride (6 [40.0]) Domperidone (2 [13.3]) Prucalopride (2 [13.3])
B – Blood and blood forming organs	6	Dabigatran (3 [50.0]) Ticagrelor (2 [33.3]) Desirudin (1 [16.7])
C – Cardiovascular system	61	Valsartan with Sacubitril (7 [11.5]) Ivabradine (5 [8.2]) Simvastatin (4 [6.6])
D – Dermatologicals	1	Isotretinoin (1 [100.0])
G – Genito-urinary system and sex hormones	5	Danazol (2 [40.0]) Darifenacin (1 [20.0]) Avanafil (1 [20.0])
J – Antiinfectives for systemic use	72	Rifampicin (4 [5.6]) Posaconazole (4 [5.6]) Fusidic acid (4 [5.6])
L – Antineoplastic and immunomodulating agents	16	Ciclosporin (8 [50.0]) Sirolimus (2 [12.5]) Irinotecan (2 [12.5])
M – Musculo-skeletal systems	2	Tizanidine (1 [50.0]) Colchicine (1 [50.0])
N – Nervous systems	47	Pimozide (8 [17.0]) St. John’s Wort (6 [12.8]) Levodopa (3 [6.4])
P – Antiparasitic systems	3	Halofantrine (2 [66.7]) Mefloquine (1 [33.3])
R – Respiratory system	22	Astemizole (6 [27.3]) Terfenadine (6 [27.3]) Mizolastine (5 [22.7])
V – Various	4	Cobicistat (3 [75.0]) Methylthioninium chloride (1 [25.0])
Total	254	

### Non-operationalizable contraindicated risk situations

Contraindicated risk situations were primarily rated non-operationalizable based on the categories ‘hypersensitivity to the respective drug’ (*n* = 820, 32.2%), ‘not operationalizable due to additional conditions’ (*n *= 635, 24.9%), and ‘no suitable coding available’ (*n *= 382, 15.0%). Supplementary Table S1 provides a comprehensive overview of the reasons for rating contraindicated risk situations as non-operationalizable.

After completion of the INTERPOLAR study, the operationalized and coded contraindication lists in the version presented in this publication are made publicly available via Zenodo (10.5281/zenodo.19483243). This version reflects their status at the time of publication and does not include any subsequent refinements based on the conduct or results of the study. Any such updates will be made available in a dedicated GitHub repository and are outside the scope of this publication: https://github.com/medizininformatik-initiative/INTERPOLAR.

## Discussion

Through extraction and operationalization of contraindications from SmPCs of the most prescribed drugs in Germany, in this work, we provide publicly available, reproduceable, and machine-readable contraindication lists for integration into CDSS and real-world data analysis. By leveraging common medical coding systems, we provide a directly applicable, highly specific knowledge base to improve medication safety that goes beyond conventional drug-drug interaction alerts in CDSS. Furthermore, we categorized and analyzed the extracted contraindications to provide guidance, which clinical conditions or drugs might be of particular importance in clinical practice.

Our analysis showed that, despite the fact that many CDSS focus on drug-drug interactions and recommendations regarding dosing in renal insufficiency, the majority of contraindications listed in SmPCs are drug-clinical condition contraindications. Across all four contraindication categories, the most frequently listed contraindicated risk situations were ‘severe liver insufficiency’, ‘pregnancy’, ‘shock’, and ‘MAO-inhibitors’. These findings suggest that clinicians should be particularly alert when prescribing or reviewing patients with such conditions or drugs. However, for confirmation and prior to clinical implementation, studies investigating the real-world prevalence and clinical relevance of these contraindications are necessary.

Our analysis also showed that the most common risk situations only account for a minority of all operationalized risk situations (e.g., the ten most frequently listed risk situations comprise only 19.1% of all operationalized risk situations). This challenges the feasibility of focusing on patients with a small subset of selected contraindications. By providing operationalized and machine-readable contraindication lists that can be directly implemented in CDSS, our work enables comprehensive alerting for all our operationalized contraindications in routine clinical practice.

Undetected and unresolved contraindications pose substantial risks for medication safety [2–4]. Previous studies have shown that, despite the high clinical relevance of contraindications, SmPCs often contain ambiguous wording regarding contraindications, leaving room for interpretation and uncertainty in clinical practice [2, 14, 25]. Since SmPCs are commonly consulted by clinicians when seeking information on drug therapy, this poses a significant issue in clinical practice. Following that, despite the implementation of various strategies, such as CDSS and medication reviews, contraindications persist as a considerable challenge in routine care and may therefore require more targeted interventions. To test their potential benefit, the contraindication lists presented in this publication will be applied within the INTERPOLAR study of the German Medical Informatics Initiative [12, 13]. This ongoing, stepped-wedge cluster-randomized multi-center study, conducted in 14 German university hospitals, compares the ‘usual care’ of ward pharmacists with an IT-supported ‘medication review’ utilizing the contraindication lists provided in this work. Therefore, INTERPOLAR aims to evaluate the additional benefit of systematic contraindication detection, which could then potentially be translated into routine clinical care. Thereby, INTERPOLAR generates a comprehensive dataset on prevalence and clinical relevance of the operationalized contraindications, taking into account clinical pharmacist feedback on CDSS alerts.

Next to the application in the INTERPOLAR study, our work could contribute to close an important clinical knowledge gap: Despite their unquestionable clinical relevance, comprehensive analyses of contraindication prevalence in both clinical and ambulatory care are still scarce in literature. Most studies addressing medication safety by leveraging real-world data focus on adverse drug events or drug-drug interactions [26–28]. The hereby presented machine-readable contraindication lists could enable systematic analyses of contraindication prevalences and potentially associated determining factors (e.g., age or polypharmacy) within any structured real-world data source such as claims data or medical registries. Additionally, aspects relevant to both medication safety and pharmacoeconomics could be evaluated (e.g., the resolution rate of contraindications and outcomes of non-resolved contraindications, such as hospitalizations).

In comparison to most CDSS, which only rarely disclose their underlying data sources, a major strength of this work is its foundation in transparency achieved by the use of publicly available SmPCs. This approach facilitates reproducibility and allows future refinement of the workflow. For instance, the workflow and the generated contraindication lists can serve as a basis for a future artificial intelligence (AI)-based contraindication extraction and operationalization workflow.

However, several limitations of this study have to be considered. First, the generated contraindication lists encompass the data available at the time of their creation. Since clinical knowledge grows continuously and SmPCs are subject to frequent updates, the lists require continuous maintenance to ensure comprehensibility. Second, the extraction and operationalization of contraindications were performed manually by various researchers. Despite the precise definition of each step within the workflow and independent double and triple verification, some contraindications in the SmPCs might be missing or coded incorrectly. Third, about half of the contraindications reported in SmPCs had to be excluded from our lists, as they were not considered codable based on our aim of ensuring high specificity when applying the contraindication lists. In particular, this applied to clinical conditions with ambiguous terms such as ‘clinically relevant’. For their accurate operationalization, additional expert knowledge or a comprehensive review of clinical data would be required, which was beyond the scope and aim of this project, but also reflects a general problem in the current presentation of contraindications in SmPCs: When contraindications are poorly defined, they become difficult to operationalize and leave clinicians vulnerable to misinterpretations that may result in medication errors or patient harm. Therefore, considering that alert fatigue represents a major concern in clinical practice when using CDSS, we intentionally prioritized specificity over sensitivity to ensure that the generated contraindication lists provide clinicians with alerts of potentially higher actionable relevance [9, 10]. Fourth, the applicability of the contraindication lists, both in the inpatient and outpatient setting, is object to the quality of coding of conditions, drugs and laboratory values, which might lead to under-reporting of contraindications in cases of insufficient coding quality and comprehensiveness. Fifth, our data set was limited to only a fraction of the available drugs in Germany and, thus, does not constitute a complete reflection of the market. However, due to the fact that this work is based on a large number of the most prescribed drugs in Germany, the generalizability of key observations can be assumed.

In conclusion, we extracted and operationalized contraindications of the most prescribed drugs in Germany, utilizing commonly used medical coding systems and categorized them according to their frequencies in the analyzed SmPCs. Thereby, we created machine-readable lists of contraindications, i.e., drug-clinical condition, drug-kidney, drug-drug group, and drug-drug, that can be implemented in CDSS or leveraged for real-world data analysis. These lists and the open-source workflow may facilitate broad application and help reduce avoidable medication-related harm by preventing contraindicated prescribing.

## Supplementary Information

Below is the link to the electronic supplementary material.

Supplementary Material 1

## Data Availability

After completion of the INTERPOLAR study, the operationalized and coded contraindication lists in the version presented in this publication are made publicly available via Zenodo (10.5281/zenodo.19483243). This version reflects their status at the time of publication and does not include any subsequent refinements based on the conduct or results of the study. Any such updates will be made available in a dedicated GitHub repository and are outside the scope of this publication: https://github.com/medizininformatik-initiative/INTERPOLAR.

## References

[1] EMA, A Guideline On Summary Of Product Characteristics (SmPC) (2009) online available at: https://health.ec.europa.eu/system/files/2016-11/smpc_guideline_rev2_en_0.pdf. accessed 01.04.2026

[2] Andrikyan W, Sponfeldner MI, Jung-Poppe L et al (2025) Physicians’ and pharmacists’ perspective on clarity and clinical relevance of absolute contraindications in summaries of product characteristics. Br J Clin Pharmacol 91:829–840. 10.1111/bcp.16331 39511858 10.1111/bcp.16331 PMC11862801

[3] Dormann H, Criegee-Rieck M, Neubert A et al (2003) Lack of awareness of community-acquired adverse drug reactions upon hospital admission: dimensions and consequences of a dilemma. Drug Saf 26:353–362. 10.2165/00002018-200326050-00004 12650635 10.2165/00002018-200326050-00004

[4] Shastay A (2017) The absence of a drug-disease interaction alert leads to a child’s death. Home Healthc Now 35:285. 10.1097/NHH.0000000000000533 28471797 10.1097/NHH.0000000000000533

[5] Kuperman GJ, Bobb A, Payne TH et al (2007) Medication-related clinical decision support in computerized provider order entry systems: a review. J Am Med Inf Assoc 14:29–40. 10.1197/jamia.M2170 10.1197/jamia.M2170 PMC2215064 17068355

[6] Shahmoradi L, Safdari R, Ahmadi H et al (2021) Clinical decision support systems-based interventions to improve medication outcomes: a systematic literature review on features and effects. Med J Islam Repub Iran 35:27. 10.47176/mjiri.35.27 34169039 10.47176/mjiri.35.27 PMC8214039

[7] Sutton RT, Pincock D, Baumgart DC et al (2020) An overview of clinical decision support systems: benefits, risks, and strategies for success. Npj Digit Med 3:17. 10.1038/s41746-020-0221-y 32047862 10.1038/s41746-020-0221-y PMC7005290

[8] Tawadrous D, Shariff SZ, Haynes RB et al (2011) Use of clinical decision support systems for kidney-related drug prescribing: a systematic review. Am J Kidney Dis 58:903–914. 10.1053/j.ajkd.2011.07.022 21944664 10.1053/j.ajkd.2011.07.022

[9] Seidling HM, Phansalkar S, Seger DL et al (2011) Factors influencing alert acceptance: a novel approach for predicting the success of clinical decision support. J Am Med Inf Assoc 18:479–484. 10.1136/amiajnl-2010-000039 10.1136/amiajnl-2010-000039 PMC3128393 21571746

[10] Bauer J, Busse M, Koch S et al (2025) Clinical–pharmaceutical assessment of medication CDSS alerts: content appropriateness and patient relevance in clinical practice. Front Pharmacol 16:1510425. 10.3389/fphar.2025.1510425 40124781 10.3389/fphar.2025.1510425 PMC11925916

[11] Kwan JL, Lo L, Ferguson J et al (2020) Computerised clinical decision support systems and absolute improvements in care: meta-analysis of controlled clinical trials. BMJ 370:m3216. 10.1136/bmj.m3216 32943437 10.1136/bmj.m3216 PMC7495041

[12] Loeffler M, Maas R, Neumann D et al (2024) INTERPOLAR – prospektive, interventionelle Studien im Rahmen der Medizininformatik-Initiative zur Verbesserung der Arzneimitteltherapiesicherheit in der Krankenversorgung. Bundesgesundheitsblatt Gesundheitsforschung Gesundheitsschutz 67:676–684. 10.1007/s00103-024-03890-w 38750238 10.1007/s00103-024-03890-w PMC11166858

[13] Neumann D, Staeubert S, Struebing A et al (2025) INTERPOLAR_MI - a study platform concept for IT-supported drug therapy safety research. Stud Health Technol Inf 327:1255–1259. 10.3233/SHTI250599 10.3233/SHTI250599 40380703

[14] Weisbach L, Schuster AK, Hartmann M et al (2023) Inconsistencies of absolute drug–drug contraindication reports: analysis of summaries of product characteristics of commonly prescribed drugs. Br J Clin Pharmacol 89:2552–2560. 10.1111/bcp.15730 37002812 10.1111/bcp.15730

[15] ICD-10-GM classification, German National Association of Statutory Health Insurance Physicians online available at: https://icd.kbv.de/icdbrowser/main.xhtml. accessed 01.04.2026

[16] ATC-Classification, German Federal Institute for Drugs and Medical Devices online available at: https://www.bfarm.de/DE/Kodiersysteme/Klassifikationen/ATC/_node.html. accessed 01.04.2026

[17] OPS-Classification, German Federal Institute for Drugs and Medical Devices online available at: https://www.bfarm.de/DE/Kodiersysteme/Klassifikationen/OPS-ICHI/OPS/_node.html. accessed 01.04.2026

[18] Kidney Disease: Improving Global Outcomes (KDIGO) CKD Work Group (2024) KDIGO 2024 Clinical Practice Guideline for the Evaluation and Management of Chronic Kidney Disease. Kidney Int 105:S117–S314. 10.1016/j.kint.2023.10.018 38490803 10.1016/j.kint.2023.10.018

[19] Flockhart DA, Oesterheld JR (2000) Cytochrome P450-mediated drug interactions. Child Adolesc Psychiatr Clin N Am 9:43–76. 10.1016/S1056-4993(18)30135-4 10674190

[20] CredibleMeds online available at: https://www.crediblemeds.org/. accessed 01.04.2026

[21] Morrissey K, Wen C, Johns S et al (2012) The UCSF-FDA TransPortal: a public drug transporter database. Clin Pharmacol Ther 92:545–546. 10.1038/clpt.2012.44 23085876 10.1038/clpt.2012.44 PMC3974775

[22] Donnelly M, Fang L, Madabushi R et al (2025) Narrow therapeutic index drugs: FDA experience, views, and operations. Clin Pharmacol Ther 117:116–129. 10.1002/cpt.3460 39529254 10.1002/cpt.3460

[23] LiverTox (2012) clinical and research information on drug-induced liver injury. National Institute of Diabetes and Digestive and Kidney Diseases, Bethesda (MD) 31643176

[24] Wermund AM, Haerdtlein A, Fehrmann W et al (2025) Drug-event pairs as indicators for the detection of adverse drug reactions during hospitalization in routinely collected electronic data sources. Clin Pharmacol Ther 117:1811–1819. 10.1002/cpt.3635 40099752 10.1002/cpt.3635 PMC12087692

[25] Weisbach L, Schuster AK, Hartmann M et al (2022) Inconsistencies and ambiguities in liver-disease-related contraindications—a systematic analysis of SmPCs/PI of major drug markets. *J Clin Med* 11:1933. 10.3390/jcm11071933 10.3390/jcm11071933 PMC9000103 35407541

[26] Sánchez-Valle J, Correia RB, Camacho-Artacho M et al (2024) Prevalence and differences in the co-administration of drugs known to interact: an analysis of three distinct and large populations. BMC Med 22:166. 10.1186/s12916-024-03384-1 38637816 10.1186/s12916-024-03384-1 PMC11027217

[27] Schechner M, Rottenkolber M, Weglage C et al (2026) Implied ADR-admissions: a cohort study introducing a novel administrative data approach for identifying drug-related hospitalisations. Drug Saf 49:337–351. 10.1007/s40264-025-01614-w 40956484 10.1007/s40264-025-01614-w PMC12924847

[28] Assiri GA, Shebl NA, Mahmoud MA et al (2018) What is the epidemiology of medication errors, error-related adverse events and risk factors for errors in adults managed in community care contexts? a systematic review of the international literature. BMJ Open 8:e019101. 10.1136/bmjopen-2017-019101 29730617 10.1136/bmjopen-2017-019101 PMC5942474

