# Supplementary material for: Development and analysis of a SmPC-based machine-readable dataset of contraindications for clinical decision support and real-world data analysis
Source: Eur J Clin Pharmacol. 2026 Jul 16;82(8):209. doi: 10.1007/s00228-026-04142-3 (PMC13375740; doi:10.1007/s00228-026-04142-3)
Supplement: Supplementary file 1 — Supplementary Material 1 [file 228_2026_4142_MOESM1_ESM.pdf]

European Journal of Clinical Pharmacology

Supplementary Material

Development and analysis of a SmPC-based machine-readable dataset of contraindications for clinical decision support and real-world data analysis

Miriam Schechner[1], Wahram Andrikyan[2,3], Thomas Bauerdick[4], Theresa Terstegen[5], Louisa Redeker[6], Martin Federbusch[7,8], Katrin Farker[9], Alexander Strübing[10], Florian Schmidt[10], Petra Thürmann[6], Sven Schmiedl[6], Hanna M. Seidling[5], Tobias Dreischulte[1], Daniel Neumann[10], Markus Loeffler[10], Renke Maas[2,11], Ulrich Jaehde[4], Martin F. Fromm[2,11] and Michael I. Sponfeldner[2,*]

for the INTERPOLAR project of the German Medical Informatics Initiative

[1] Institute of General Practice and Family Medicine, LMU University Hospital, LMU Munich, Munich, Germany.

[2] Institute of Experimental and Clinical Pharmacology and Toxicology, Friedrich-Alexander-Universität Erlangen-Nürnberg, Erlangen, Germany.

[3] Universitätsklinikum Erlangen, Erlangen, Germany.

[4] Department of Clinical Pharmacy, Institute of Pharmacy, University of Bonn, Bonn, Germany.

[5] Heidelberg University, Medical Faculty Heidelberg / Heidelberg University Hospital, Internal Medicine IX, Clinical Pharmacology and Pharmacoepidemiology, Cooperation Unit Clinical Pharmacy, Im Neuenheimer Feld 410, 69120 Heidelberg, Germany.

[6] Department of Clinical Pharmacology, School of Medicine, Faculty of Health, Witten / Herdecke University, Wuppertal, Germany.

[7] Department for Clinical AI and Translational Medicine, University of Leipzig Medical Center, Leipzig, Germany.

[8] Institute of Laboratory Medicine, Clinical Chemistry and Molecular Diagnostics, University of Leipzig Medical Center, Leipzig, Germany.

[9] Institute for Hospital Pharmacy, University Center for Pharmacotherapy and Pharmacoeconomics, Jena University Hospital, Jena, Germany.

[10] Institute for Medical Informatics, Statistics and Epidemiology (IMISE), Leipzig University, Leipzig, Germany.

[11] FAU NeW – Research Center New Bioactive Compounds, Friedrich-Alexander-Universität Erlangen-Nürnberg, Erlangen, Germany.

* Corresponding author: Michael I. Sponfeldner, Institute of Experimental and Clinical Pharmacology and Toxicology, Friedrich-Alexander-Universität Erlangen-Nürnberg, Fahrstr. 17, 91054 Erlangen, Germany; Tel: +49 9131 85 22578; Fax: +49 9131 85 22773; E-mail: michael.sponfeldner@fau.de

SUPPLEMENTARY MATERIAL

1. **Supplementary Methods**
2. **Supplementary Figure S1 Characterization of the 688 most prescribed drugs in Germany according to Weisbach et al.**
3. **Supplementary Table S1 Classification of non-operationalizable contraindicated risk situations**

Supplementary Methods

Selection of Summaries of Product Characteristics (SmPCs)

The selection of drugs used for the analyses is based on a prior study by Weisbach et al. [1], which identified the most prescribed drugs in the German outpatient and inpatient sector (n=688). The selection by Weisbach et al. [1] was formed based on the TOP 1000 drugs of two German university hospitals (inpatient care) as well as the TOP 1000 drugs according to the German drug prescription report (outpatient care). From these lists, duplicates, medicinal devices, nutrition and inhouse formulas were removed, resulting in a list of 688 drugs. At first, SmPCs were considered eligible for extraction of contraindications, if they met both of the following criteria: 1) the SmPC was issued by the original manufacturer of the drug and 2) the SmPC or an updated version was published within the last 5 years (cut-off: July 2019). In addition, if multiple dosages were available, the SmPC corresponding to the highest available dosage was selected. The selection process of SmPCs followed a hierarchical approach: First, the database fachinfo.de [2] was searched for eligible SmPCs. If no appropriate SmPC could be identified, the database gelbe-liste.de [3] was consulted. If still no SmPC was eligible, the BfArM-AMIce database [4] was searched.

If no eligible SmPC, according to the criteria mentioned above, could be identified across all three databases, the stepwise search strategy was repeated to identify a SmPC from a generic manufacturer issued within the last 5 years with the requirement of having the same indication and route of application. In cases where this search also retrieved no eligible SmPC, the most recent SmPC, irrespective of manufacturer, was selected for contraindication extraction. The selection of SmPCs was performed in July 2024.

Operationalization of Anatomical Therapeutic Chemical (ATC) codes of the 688 drugs most commonly prescribed in Germany

For the 688 drugs selected for operationalization, a ‘primary’ ATC code was assigned, based on the ATC code provided by the respective SmPC. In addition to the primary ATC code, all other relevant, secondary ATC codes referring to the same active agent were identified via the official database operated by the German Federal Institute for Drugs and Medical Devices (BfArM.de, ATC Version 2026 [5]). They were grouped by route of administration, i.e., systemic, dermal, ophthalmologic, inhalative, and other. For drugs available as single-agent product as well as fixed-dose combination drug, e.g., ramipril and hydrochlorothiazide, all ATC codes containing the respective drug were considered. In cases where both a single-agent product and a fixed-dose combination containing that agent were part of the most prescribed drugs in Germany, duplicate ATC codes were removed.

Operationalization of contraindications within the drug-clinical condition list based on Logical Observation Identifiers Names and Codes (LOINC)

LOINC codes were used for coding of clinical conditions through specific laboratory parameters, e.g., for the clinical condition ‘hyperkalaemia’, based on the following methodology:

Prior to evaluation of operationalizability of risk situations through LOINC, all drug-clinical condition contraindications were screened based on the feasibility of coding through one specific LOINC code, i.e., risk situations that required multiple different abnormal laboratory parameters were also deemed non operationalizable. In general, one primary LOINC was used for coding for a risk situation in the drug-clinical condition

contraindication list. These primary LOINCs will subsequently be used as proxy for a set of multiple secondary suitable LOINCs, which will be published in near future. After identification of operationalizable risk situations and coding through primary LOINCs, multiple strategies for choosing adequate laboratory parameter cut-off values were used:

1) For clinical conditions referring directly to a specific laboratory parameter without a specific cut-off mentioned, i.e., hyperkalaemia, the respective lower limit of normal / upper limit of normal was used.

2) For clinical conditions referring directly to a specific laboratory parameter with a specific cut-off mentioned, i.e., leukopenia (> 2.6 x 10^9 WBC/L), the specific cut-off was used.

3) For clinical conditions referring directly to a specific laboratory parameter with an additional qualifier, i.e., severe hyperkalaemia, a literature search was performed to define the most commonly used cut-off.

4) For clinical conditions which can be described by certain laboratory parameters (e.g., ‘icterus’) a literature search was carried out to define the most commonly used cut-off for this condition.

For this study the file Loinc.csv from the LOINC database 2.75 was used, which is freely available (https://loinc.org/downloads/archive/).

Machine readability of contraindication lists and validation

Machine readability of the extracted information is achieved through tabular structures in which each row represents a single contraindication. Columns contain either individual codes or space-separated lists of codes with corresponding descriptive texts. All tables

include the ATC code and the generic name of the drug that may be contraindicated in combination with a specific risk situation. In the drug–drug list, the contraindicated drug is represented by another ATC code and its corresponding name. The drug-drug group list comprises ATC codes sets representing the contraindicated drug groups. The drug-clinical condition list additionally includes LOINC, OPS, and ICD-10 codes together with their descriptions. The drug–kidney list, representing a subcategory of drug–clinical condition contraindications, follows an analogous structure. Technical validation procedures were applied to ensure that the manually extracted and extensive lists contain only structurally valid and existing codes. Furthermore, the code combinations do not overlap across list boundaries. This prevents double counting and allows all lists to be used simultaneously in clinical decision support systems (CDSS) without generating multiple alerts of different origins for equivalent contraindications.

Characterization of the most prescribed drugs in Germany

The 688 most prescribed drugs in the German inpatient and outpatient sector, as identified by Weisbach et al. [1], were characterized according to the ATC Classification 2026. The distribution of drug categories at ATC level 1 (e.g., ‘A’ corresponding to ‘Alimentary Tract and Metabolism’) and 2 (e.g., ‘A04’ corresponding to ‘Antiemetics and Antinauseants’) is shown in *Supplementary Figure S1*. At ATC level 1, the most prescribed drug categories were ‘Nervous System’ (n=144, 20.9%), ‘Alimentary Tract and Metabolism’ (n=105, 15.3%), and ‘Cardiovascular System’ (n=94, 13.7%). At ATC level 2, the leading drug categories were ‘Antibacterials for systemic use’ (n = 44, 6.4%), ‘Psycholeptics’ (n=38, 5.5%), and ‘Ophthalmologicals’ (n=33, 4.8%).

Supplementary Figure S1 Characterization of the 688 most prescribed drugs in Germany according to Weisbach et al.

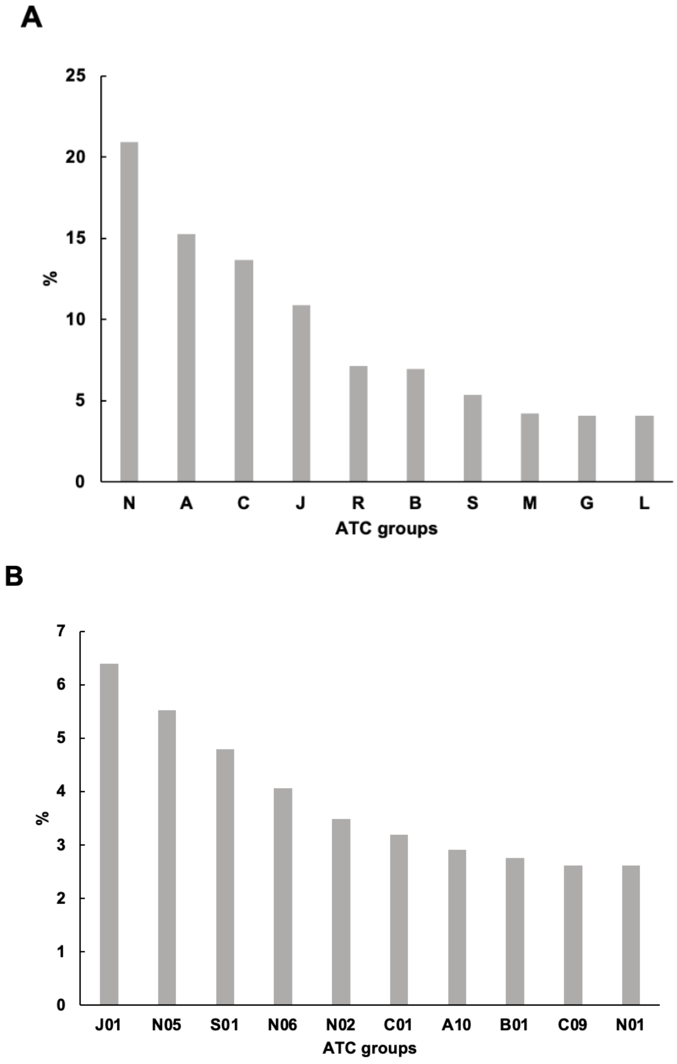

Characterization of the most prescribed drugs in Germany at ATC level 1 (Figure 2A) and ATC level 2 (Figure 2B). The list of the most prescribed drugs is based on Weisbach et al. [1]. Abbreviations: ATC = Anatomical Therapeutic Chemical; A = Alimentary tract and metabolism; A10 = Drugs used in diabetes; B = Blood and blood forming organs; B01 = Antithrombotic agents; C = Cardiovascular system; C01 = Cardiac therapy; C09 = Agents acting on the renin-angiotensin systm; G = Genito urinary system and sex hormones; J = Antiinfective for systemic use; J01 = Antibacterials for systemic use; L = Antineoplastic and immunomodulating agents; M = Musculo-skeletal system; N = Nervous system; N01 = Anesthetics; N02 = Analgesics; N05 = Psycholeptics; N06 = Psychoanaleptics; R = Respiratory system; S = Sensory organs; S01 = Ophthalmologicals

Supplementary Table S1 Classification of non-operationalizable contraindicated risk situations

**Non-operationalizable contraindicated risk situation due to the following reasons…**	**n** *(% of all non-operationalizable contraindicated risk situations)*
**Drug-clinical condition**	
Hypersensitivity to the respective drug[a]	820 *(32.2)*
Not Operationalizeable Due to Additional Conditions[b]	579 *(22.7)*
No Suitable Coding Available[c]	382 *(15.0)*
Contraindicated Risk Situation Too Unspecific[d]	274 *(10.8)*
Dosage Form Restriction[e]	113 *(4.4)*
Acute Contraindicated Risk Situation[f]	111 *(4.4)*
Duplicate Entry (Identical ATC Code)[g]	46 *(1.8)*
ATC Code Too Unspecific[h]	43 *(1.7)*
Time-Based Restriction[i]	35 *(1.4)*
Dose Restriction[j]	11 *(0.4)*
Weight Restriction[k]	3 *(0.1)*
Age Restriction[l]	2 *(0.1)*
Other Reasons[m]	1 *(0.0)*
**Total**	2420 *(95.0)*
**Drug-kidney**	
Not Operationalizeable Due to Additional Conditions[b]	14 *(0.6)*
Contraindicated Risk Situation Too Unspecific[d]	6 *(0.2)*
Dose Restriction[j]	5 *(0.2)*
**Total**	25 *(1.0)*
**Drug-drug group**	
Group Definition Not Possible[n]	12 *(0.5)*
Not Operationalizeable Due to Additional Conditions[b]	10 *(0.4)*
Dosage Form Restriction[e]	7 *(0.3)*
Duplicate Entry (Identical ATC Code)[g]	6 *(0.2)*
Dose Restriction[j]	4 *(0.2)*
Time-Based Restriction[i]	2 *(0.1)*
**Total**	41 *(1.6)*
**Drug-drug**	
Not Operationalizeable Due to Additional Conditions[b]	32 *(1.3)*
Dose Restriction[j]	10 *(0.4)*
Dosage Form Restriction[e]	8 *(0.3)*
Time-Based Restriction[i]	4 *(0.2)*
Not Mappable Due to Identical ATC Code[o]	3 *(0.1)*
No ATC Code Available[p]	3 *(0.1)*
Medication Too Unspecific[q]	1 *(0.0)*
**Total**	61 *(2.4)*
**Overall Total**	2547 *(100.0)*

[a] e.g., Hypersensitivity to methotrexate; [b] The contraindication depends on one or more additional qualifying conditions and therefore could not be operationalized (e.g., pacemaker dependency, i.e., the heart rate is generated *exclusively* by the pacemaker); [c] The condition is not representable within the used coding systems (ICD-10, OPS, ATC, LOINC) due to the absence of an appropriate or sufficiently specific code (e.g., Torsades de pointes); [d] The operationalization of such contraindications would have required additional expert consensus (e.g., clinically relevant bleeding); [e] The contraindication is restricted to certain dosage forms (e.g., subcutaneous administration); [f] Acute, transient condition not reliably capturable in routine coded data (e.g., acute opioid intoxication); [g] Entries sharing the same ATC code were considered duplicates and consolidated, regardless of formulation or route of administration (e.g., amiodarone solution for injection vs. tablets); [h] The ATC code of the considered drug lacks sufficient specificity to reliably identify the products to which the contraindication applies (e.g., ATC code A03FP30 – combinations); [i] The contraindication applies only within a certain time period (e.g., during childbirth); [j] The contraindication is restricted to a certain dosage (e.g., Hypothyroidism with a rosuvastatin dose > 40 mg/day); [k] The contraindication is restricted to a certain body weight (e.g., body weight < 35 kg); [l] The contraindication is restricted to a certain age (e.g., patients aged 60 years or older); [m] e.g., women; [n] No standardized or reliable source available to define the drug group (e.g., mercury-containing drugs); [o] The corresponding drug is contraindicated with itself (e.g., bupropion is contraindicated with other bupropion-containing drugs); [p] No suitable ATC code available (e.g. alcohol); [q] Non-specific or ambiguous substance term in Summary of Product Characteristics (e.g., phenothiazine)

References

1. Weisbach L, Schuster AK, Hartmann M, et al. (2023) Inconsistencies of absolute drug–drug contraindication reports: Analysis of Summaries of Product Characteristics of commonly prescribed drugs. *Br J Clin Pharmacol* 89:2552–2560. https://doi.org/10.1111/bcp.15730

2. fachinfo.de. online available at: https://www.fachinfo.de/. accessed 01.04.2026

3. Gelbe Liste Pharmindex. online available at: https://www.gelbe-liste.de. accessed 01.04.2026

4. AMICe drug information system, German Federal Institute for Drugs and Medical Devices. online available at: https://www.bfarm.de/DE/Arzneimittel/Arzneimittelinformationen/Arzneimittel-recherchieren/AMIce/_node.html. accessed 01.04.2026

5. ATC-Classification, German Federal Institute for Drugs and Medical Devices, online available at:

https://www.bfarm.de/DE/Kodiersysteme/Klassifikationen/ATC/_node.html, accessed 01.04.2026
